# Electricity Intensity of Internet Data Transmission: Untangling the Estimates

**DOI:** 10.1111/jiec.12630

**Published:** 2017-08-01

**Authors:** Joshua Aslan, Kieren Mayers, Jonathan G. Koomey, Chris France

**Affiliations:** 1 Center for Environmental Strategy, Faculty of Engineering and Physical Sciences, University of Surrey, Guildford, Surrey, GU2 7XH UK; 2 Social Innovation Center, INSEAD, Fontainbleau, France; 3 Sony Interactive Entertainment Europe, London, UK; 4 School of Earth, Energy, & Environmental Sciences, Stanford University, Burlingame, CA USA

**Keywords:** electricity intensity, energy, industrial ecology, information and communication technology (ICT), Internet, meta-analysis

## Abstract

In order to understand the electricity use of Internet services, it is important to have accurate estimates for the average electricity intensity of transmitting data through the Internet (measured as kilowatt-hours per gigabyte [kWh/GB]). This study identifies representative estimates for the average electricity intensity of fixed-line Internet transmission networks over time and suggests criteria for making accurate estimates in the future. Differences in system boundary, assumptions used, and year to which the data apply significantly affect such estimates. Surprisingly, methodology used is not a major source of error, as has been suggested in the past. This article derives criteria to identify accurate estimates over time and provides a new estimate of 0.06 kWh/GB for 2015. By retroactively applying our criteria to existing studies, we were able to determine that the electricity intensity of data transmission (core and fixed-line access networks) has decreased by half approximately every 2 years since 2000 (for developed countries), a rate of change comparable to that found in the efficiency of computing more generally.

**Supplementary Information:**

The online version of this article (doi:10.1111/jiec.12630) contains supplementary material, which is available to authorized users.

## Introduction

Global Internet data traffic has increased more than fivefold since 2010 and continues to grow, with some predictions suggesting threefold growth over the next 5 years (Cisco 2015). This growth is driven by increasing number of connected devices, expected to reach 28 billion by 2020 (Ericsson 2016), and increasing use of digital and cloud-based services. For example, in 2012, consumption of online movies overtook sales of DVDs and Blu-rays in the United States, on a per-unit basis (Cryan 2012).

With rapid growth in Internet use, concern has arisen over the electricity consumption of Information and Communication Technology (ICT). It is estimated that ICT products and services accounted for 3.9% of world-wide electricity consumption in 2007, increasing to 4.6% in 2012 (Heddeghem et al. 2014). As a result, policy makers have focused attention on increasing the energy efficiency of Internet networks. For example, a recent International Energy Agency (IEA) report stated that the development of energy efficiency metrics was one of three key considerations required for effective policy making to reduce the energy use of networks (IEA 2014).

There have been several attempts to estimate the electricity intensity of Internet data transmission, which is defined as the electrical “energy consumed per amount of data transmitted” (Coroama et al. 2013, 2). Electricity intensity is a measure for assessing the efficiency of data transmission through the Internet over time. This study focuses on the average electricity intensity, rather than specific or marginal estimates, as the average has more application potential, representing the historical measure of electricity used to transmit data.

Electricity intensity of Internet data transmission is often used in life cycle assessment (LCA) research to estimate the carbon-equivalent emissions arising from Internet use. For example, Mayers and colleagues (2014) applied electricity intensity estimates as part of an LCA study comparing different methods of games distribution, concluding that the carbon-equivalent emissions arising from an Internet game download (for an average 8.8-gigabyte [GB] game) were higher than those from Blu-ray Disc distribution in 2010. Within LCA studies, electricity intensity of Internet data transmission is typically calculated as a ratio of total electricity use and total data throughput, similar to the way in which carbon emissions are allocated for transport networks and electricity generation and transmission.

Existing estimates for the electricity intensity of Internet data transmission, for 2000 to 2015, vary up to 5 orders of magnitude, ranging from between 136 kilowatt-hours (kWh)/GB in 2000 (Koomey et al. 2004) and 0.004 kWh/GB in 2008 (Baliga et al. 2009). While increased efficiency over time can account for 2 orders of magnitude of this variation (based on results presented below), alone it does not explain the spread of results. Differences in the system boundary of each study and the assumptions applied also can cause variability (Schien and Preist 2014; Coroama and Hilty 2014). Additionally, Schien and Preist (2014) suggest that the approach used can introduce a significant source of uncertainty, classified as either top-down or bottom-up:

- Top-down: Network/subsystem level total electricity consumption, divided by total data transferred through network/subsystem (summed to find total).
- Bottom-up: Sum of electricity consumption, typically at the level of individual equipment, divided by the data transferred through the equipment (often requiring application of utilization factors).

So-called top-down approaches have been criticized for overestimating electricity intensity, whereas bottom-up approaches have been considered to underestimate electricity intensity (Schien and Preist 2014). Nevertheless, there appears to be uncertainty over which estimates best reflect real-world/mean data transmission (we will refer to such estimates as “representative”).

Accurate and representative estimates for the electricity intensity of Internet data transmission are required for effective research and also for effective decision making by policy makers and industry interested in improving the energy efficiency of network technologies (IEA 2014). This study is concerned with Internet networks in developed countries, the characteristics (and therefore electricity intensity) of which tend to be more comparable across countries and better understood than networks in developing countries.

This study undertakes a meta-analysis to identify the most accurate estimates of average electricity intensity for data transmitted over the Internet to:

- Understand current approaches for estimating electricity intensity of Internet data transmission;
- Establish criteria to identify the most robust approaches and representative existing estimates; and
- Highlight potential underlying trends that may describe characteristics of Internet data transmission, for example, rapid improvements in electricity efficiency over time.

## Methodology

Electricity intensity is measured in kWh/GB or joules per bit transmitted. We reviewed 14 studies providing estimates of electricity intensity, converted them to common units of kWh/GB and then tabulated them chronologically. Average electricity intensity of transmission networks is an important metric for use in life cycle assessments evaluating the carbon emissions of Internet services. LCA studies usually depend upon average energy intensity to calculate impact of *background systems* such as in transport networks and electricity production and transmission, which are examples of *attributional allocation* approaches (EC 2010). Coroama and colleagues (2015) argue that electricity use of access networks and home/on-site networking equipment should be allocated by the time used and not data, as the electricity use does not vary with data volume. Nevertheless, Internet usage varies daily, as discussed previously, and access networks and home/on-site networking equipment are provisioned to handle peak capacity at all times. The electricity use for these subsystems is a function of both data volume and time, creating a problem on how to best allocate electricity use to different levels of Internet activity. In accordance with estimates from existing studies, data are presented in kWh/GB in order to fully account for the overall energy use of Internet data transmission in previous years.

**Figure 1:**
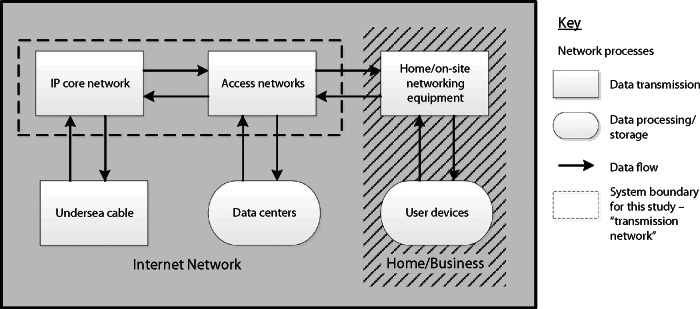
Simplified Internet structure diagram, showing scale over which key processes operate. The dotted box represents the common system boundary (for data transmission) selected for this study.

The Internet is a large and complex system, often simplified into subsystems such as in figure 1 and table 1.

**Table 1:** List of Internet subsystems with descriptions and equipment examples

*Subsystem*	*Description*	*Equipment examples*
Data centers	Buildings housing servers used to carry out a large variety of functions (e.g., e-mail, financial transactions, social media, etc.) and store data. Data centers often require air conditioning units, power supply units, and other technologies to support these computer systems. Servers within data centers can be considered as end devices, which provide services accessed via the Internet.	Servers, storage equipment, power and cooling equipment, etc.
Undersea cable	High-bandwidth cable infrastructure connecting continents and countries, often traversing very long distances. This is sometimes grouped under Internet core.	Submarine communications cable, amplifiers, etc.
IP core network	Internet Service Provider (ISP) equipment which form regional, national, and global networks. This typically includes equipment that uses Internet Protocol (IP), the principle communications protocol which allows for the routing and relaying of data across networks.	IP core/metro/edge switches and routers, transmission link elements (copper, fiber optic, radio links, etc.), and supporting infrastructure for cooling, power, etc. (Malmodin et al. 2014)
Access network	Equipment connecting subscribers (or users) to ISPs, differing from the core network, which connects servers to different ISPs.	Routers, communications cable, transmission and switching equipment, etc. (including; PSTN, xDSL, DSLAM, FTTx, CATV, etc.)
Home/on-site networking equipment	Also referred to as Customer Premise Equipment (CPE), equipment used to access the Internet and provides a link to the user’s edge device, based on the customer’s premise (e.g., in the home or office building). Often used to maintain a constant on-demand connection. Home/on-site networking equipment can also form a Local Area Network (LAN).	Routers, modems, etc.
User device	Consists of the wide range of equipment a consumer may use to draw a function from the Internet	Games consoles, PCs/laptops, smartphones, tablets, etc. Any connected device.

*Note*: PCs = personal computers.

We grouped the results by Internet subsystem (according to definitions in table 1), to evaluate the impact of differing system boundaries on variability of estimates. Across the 14 studies, estimates were derived from eight different combinations of subsystems. We therefore recalculated estimates to represent a common system boundary (see figure 1), including the Internet Protocol (IP) core network and access networks only, which we refer to as the “transmission network.” This system boundary was chosen as it represents the network of equipment used for data transmission and access at a national level. The electricity intensity of the transmission network is independent of the data type; for example, media streaming, financial transactions, e-mail, etc. The electricity intensity of user devices and data centers is highly variable, depending largely on the service being provided (Coroama et al. 2015). These subsystems, together with home/on-site networking equipment, also tend to have low utilization and high “fixed” electricity use, making estimates sensitive to assumptions on usage and the allocation method used. This approach follows the argument of Coroama and colleagues (2015), who suggest assessing user devices and data centers separately to the transmission network “and to add them up when needed—for example, for the assessment of the energy needs of a specific service” (Coroama et al. 2015, 12).

Additionally, it was not possible to separate estimates for undersea cable; we assumed therefore that removing their contribution would have minimal impact (based on Malmodin et al. [2014]). Where this is the case, we identify estimates would be slightly lower (denoted by asterisk [“*”] symbol), had undersea cable been subtracted.

The different methods used were also analyzed to see if they affected the estimates derived. In addition, the year to which the data apply, type of access networks, and technical assumptions used were analyzed to determine their influence on results. From this analysis, criteria were established for selecting representative estimates of electricity intensity for transmission networks and then applied to review estimates for each study.

## Results and Analysis

Estimates from the 14 studies are shown in table 2, ranging from Baliga and colleagues (2009) estimate of 0.004 kWh/GB for the year 2008; to the earliest identified estimate made by Koomey and colleagues (2004), 136 kWh/GB for 2000 (later corrected by Taylor and Koomey [2008] to 92 to 160 kWh/GB). These authors also provide an estimate of 9 to 16 kWh/GB for 2006, using the same methodology. By contrast, the most recent estimate for the year 2015 is 0.023 kWh/GB (Malmodin and Lundén 2016). These results do not tell the full story, however, as the system boundary differs greatly between studies; from considering the IP core network only (Malmodin et al. 2012); to several studies which included all subsystems, from data centers to user devices (Costenaro and Duer 2012; Malmodin et al. 2014).

**Table 2:** Original system boundary and published estimate for electricity intensity of Internet data transmission from relevant studies and adjusted estimates of IEI considering a common system boundary of Internet core and access networks (highlighted)

			System boundary (Internet subsystems)						Estimate (kWh/GB)	
Study		Year to which data apply	Data centers	Undersea cable	IP core network	Access networks	Home/on-site networking equipment	User device	Original system boundary	Transmission network
[1]	Koomey et al. (2004)	2000	✓		✓	✓			136	7.3^a^
[2]	Taylor and Koomey (2008)	2000	✓		✓	✓			92 to 160	6.5 to 7.1^b^
		2006							9 to 16	0.65 to 0.71^b^
[3]	Baliga et al. (2009)	2008^c^		✓	✓	✓			0.17	0.17*
		2008^d^							0.004 to 0.009	0.004* to 0.009*
[4]	Weber et al. (2010)	2008	✓		✓	✓			7	∼2.2^e^
[5]	Coroama et al. (2013)	2009		✓	✓	✓			0.2	0.2*
[6]	Williams and Tang (2012)	2010	✓		✓	✓			0.3	0.013
[7]	Malmodin et al. (2012)	2010			✓				0.08	—
[8]	Malmodin et al. (2014)	2010	✓	✓	✓	✓	✓	✓	2.48	0.16^f^
[9]	Costenaro and Duer (2012)	2011	✓	✓	✓	✓	✓	✓	5.12	0.7*
[10]	Shehabi et al. (2014)	2011			✓	✓	✓		0.29	0.11^g^
[11]	Schien and Preist (2014)	2011			✓	✓			0.02	0.02
[12]	Krug et al. (2014)	2012	✓		✓	✓	✓	✓	7.2	0.14^h^
[13]	Schien et al. (2014)	2014^i^		✓	✓				0.052	—
[14]	Malmodin and Lundén (2016)	2015	✓	✓	✓	✓	✓	✓	—	0.023^j^

*Notes*: a) Calculated based on assumptions used in Koomey and colleagues (2004), see the Supporting Information available on the Journal’s website; b) calculated based on assumptions used in Taylor and Koomey (2008), see the Supporting Information on the Web; c) estimate for low access rates; d) estimate for high access rates; e) calculated based on same assumptions used by Weber and colleagues (2010); f) estimates taken directly from Malmodin and colleagues (2014); g) calculated based on same assumptions used by Shehabi and colleagues (2014), see the Supporting Information on the Web; h) calculated based on discussions with authors from Krug and colleagues (2014), see the Supporting Information on the Web; i) assumed year in which data apply, although based on data from multiple source years; j) estimate provided by Malmodin (2016) based on data from Malmodin and Lundén (2016).

IP = Internet Protocol; kWh/GB = kilowatt-hours per gigabyte. The Asterisk [“*”] symbol denotes estimates where undersea cable could not be separated from the system boundary.

Recalculating estimates to reflect a common system boundary for transmission networks only (furthest right-hand column in table 2) reduced some estimates by up to 2 orders of magnitude. System boundary therefore has a substantial impact on the estimate for electricity intensity. Results for the transmission network system boundary range from 7.3 kWh/GB for 2000 (Taylor and Koomey 2008) to 0.004 kWh/GB for 2008 (Baliga et al. 2009). The effect of methods used, year to which the data apply, characteristics of access networks, and technical assumptions on results are evaluated in table 3.

**Table 3:** Existing research for Internet electricity use, categorized by the following; methods used; year in which data was collected; geographical scope; equipment considered; access types included; power use effectiveness (PUE), utilization factor; number of hops; change in data flow in system; change in energy use of system; change in energy intensity of system

			*Method used*				*Scope*			*Technical assumptions*			*Extrapolation assumptions*		
*Study*		*Year to which data apply*	*Model*	*AEC*	*Direct Measure*	*Extrapolation*	*Geography*	*Equipment*	*Access Networks*	*PUE*	*Utilization factor*	*No. of Hops*	*∆ Data Flow*	*∆ Energy Use*	*∆ Intensity*
[1]	Koomey et al. (2004)	2000		✓			USA	Legacy inc.	All	2.0					
[2]	Taylor and Koomey (2008)	2000 2006		✓		✓	USA	Legacy inc.	All	2.0				+14%/yr	
[3]	Baliga et al. (2009)	2008 2008	✓			✓	Global	State-of-the-art	ADSL, PON, FTTN, PtP	2.0	100%	12 to 14	+42%/yr		–20%/yr
[4]	Weber et al. (2010)	2008				✓	USA	Legacy inc.	All	1.8			+50%/yr	+14%/yr	–30%/yr
[5]	Coroama et al. (2013)	2009			✓		Specific network path	State-of-the-art	FTTN	2.0	26.3%	24			
[6]	Williams and Tang (2012)	2010	✓				UK	Specific	All	1.9	25 to 60%	12 to 24			
[7]	Malmodin et al. (2012)	2010	✓		✓		Sweden	Legacy inc.	n/a	1.8					
[8]	Malmodin et al. (2014)	2010	✓		✓	✓	Sweden	Legacy inc.	All	1.8			+30%/yr		
[9]	Costenaro and Duer (2012)	2011	✓				Global	Unknown	All	1.25 to 2.0	50 to 100%				
[10]	Shehabi et al. (2014)	2011				✓	USA	Specific	All	1.3	40%	12 to 14			–20%/yr
[11]	Schien and Preist (2014)	2011	✓			✓	Global	Legacy inc.	All	2.0		12			–12.5%/yr
[12]	Krug et al. (2014)	2012	✓		✓		UK	Legacy inc.	All	2.0		17	+25 to 30%/yr		
[13]	Schien et al. (2014)	2014	✓				Global	State-of-the-art	n/a	2.0	15 to 33%				
[14]	Malmodin and Lundén (2016)	2015	✓		✓	✓	Sweden	Legacy inc.	All						

*Note*: AEC = annual electricity consumption.

### Methods Used

We identified four different methods used across the 14 studies (shown in table 3); modeling, annual electricity consumption (AEC), direct measurements, and extrapolation.

#### Modeling

Each study in table 2 could be considered to have modeled the Internet in some way (through the need to simplify the system due to the complexity and scale of the Internet). However, here the modeling approach is a distinct method—whereby equations based on parameters such as energy consumption of equipment, usage, and data flow have been derived to describe the Internet subsystems under study (requiring specific data inputs for the equipment used). For example, Baliga and colleagues (2009) give a detailed mathematical approach to estimating the electricity intensity of Internet data transmission and derive equations for the electricity intensity of each subsystem of the Internet at different bandwidths. In this example, the input data are based on a narrow range of power consumption data for specific pieces of equipment and rely on many assumptions for the characteristics of the network and data traffic.

An advantage of modeling is that it may be used to make future predictions for electricity intensity, or can be used to estimate the impact of changes in specific variables (such as increasing bandwidth). On the other hand, such models are highly sensitive to input variable assumptions and boundary choices. The input data from Baliga and colleagues (2009) is based on the power ratings for specific pieces of equipment (which may not accurately reflect equipment in use) and many assumptions for variables such as energy efficiency and utilization, which can lead to uncertainty in results. Costenaro and Duer (2012) model the global Internet using top-down data based on Raghavan and Ma (2011), which is also heavily based on such assumptions.

Schien and Preist (2014) combine the modeling approaches of several researchers to develop a meta-model for different subsystems of the Internet (Baliga et al. 2009; Van Heddeghem et al. 2012). The model of Schien and Preist (2014) used input data and the assumptions from several preceding studies (Baliga et al. 2009; Coroama et al. 2013; Kilper et al. 2011), extrapolating to a base year of 2014 by applying an improvement rate of 12.5% per annum from Tamm and colleagues (2010). A pure modeling approach is later taken for core networks by Schien and colleagues (2014). These methods, however, are still heavily dependent on the accuracy of the assumptions used, even though the input data for equipment energy use are more comprehensive than Baliga and colleagues (2009) (e.g., using data for many different servers, rather than a few specific examples).

#### Annual Electricity Consumption

AEC uses data on the power consumption, usage, and the stock of existing equipment within a network to estimate total energy used over a period. This approach typically uses estimates for annual electricity consumption of equipment and divides by estimated annual data traffic for the corresponding equipment. This is the approach taken by Koomey and colleagues (2004), which has been wrongfully categorized as a top-down approach in previous articles. Koomey and colleagues (2004) use AEC data for network equipment from Roth and colleagues (2002) and divide these data by estimates for annual data flows. This is the earliest attempt at estimating the electricity intensity of transmission networks found in the peer-reviewed literature. The advantage of using AEC data over the modeling approaches described above is that it requires fewer assumptions and can provide a more accurate representation (provided AEC data are accurate). For example, assumptions for utilization factor are not required as they are implicit in these estimates.

#### Direct Measurement

Another approach is to directly measure the power consumption and data traffic of equipment within a network. The study by Coroama and colleagues (2013) is based on measurements of electricity use from equipment employed within the specific data path for a single teleconference event. This electricity use was then divided by the data transfer rate for the teleconference (40 megabits per second) and multiplied by the time period of the event to determine the electricity intensity of the network used for the teleconference. Coroama and colleagues (2013) case study estimate of 0.2 kWh/GB is put forward as “pessimistic” and the authors go on to state “that the global average for the transmission electricity intensity must be smaller than 0.2 kWh/GB” (Coroama et al. 2013, 6).

It is unlikely that a case study based on a specific network path for a teleconference between Japan and Sweden can be used as the basis of a representative average for transmission network electricity intensity. Although the study is concerned with data transmission equipment, the range of different types of equipment used within a country-wide network is far greater than those measured by Coroama and colleagues (2013). The advantage of direct measurement is that it will always lead to more accurate estimate than a modeled estimate. Taking direct measurements for all equipment within the network, however, is often infeasible due to the dynamic scale and complexity of the Internet.

#### Extrapolation

Finally, some researchers extrapolate existing estimates, by applying factors for changes in energy use of equipment or data traffic, to derive an estimate for a different base year. Shehabi and colleagues (2014) derive their estimate of electricity intensity for 2011 by applying an energy efficiency improvement factor to the 2009 and 2010 based estimates made by Coroama and colleagues (2013) and Malmodin and colleagues (2014) respectively, then extrapolating. They apply a 20% improvement rate, taken from Malmodin and colleagues (2014). The danger with this approach is that the accuracy of extrapolations is strongly dependent on the accuracy of the original estimates, as well as that of the assumed rates of change for the projection. The complexities of such approaches are discussed further below.

#### Combined Approaches

Several researchers combine different approaches. Malmodin and colleagues’ (2014) estimate is made up of both empirical data, with access to organizational data from Swedish Internet Service Provider (ISP) TeliaSonera, and energy measurements for several thousand network sites. Malmodin and colleagues (2014) also developed energy consumption models based on supplier energy use information comprising a database of hundreds of thousands of network equipment entities, which was aggregated and compared to the value obtained from the site-level analysis (the same method is used by Malmodin and Lundén [2016], who update their 2012 estimate for 2015).

Krug and colleagues (2014) similarly present an organizational model of network electricity use of the UK ISP, BT, based on power measurements of sample equipment. The advantage of combined approaches over that of Baliga and colleagues (2009) is that Krug and colleagues (2014), Malmodin and colleagues (2014), and Malmodin and Lundén (2016) are able to base these models on inventories of actual equipment in use to represent the network, as well as using organizational site-level data to corroborate estimates. They also use measurements of total network data flows.

Previous research has suggested that top-down and bottom-up approaches lead to over- and underestimations of results, respectively. We found these classifications to be limiting as they do not explain the actual methods used. Furthermore, the method used is not a major cause of variability in estimates. In fact, a combination of methods can be used to verify estimates, as observed by Krug and colleagues (2014, 2): “an advantage of our study is that we can use the top-down analysis to verify a bottom-up analysis based on deployed equipment.” In addition, the use of modeling and extrapolation approaches without data validation must rely on assumptions, which can have higher uncertainty and therefore data availability can be more limiting with these methods.

More important than method used is the scale of network considered; the studies in table 3 have either focused on specific networks or network paths (e.g., Coroama et al. 2013), national-level networks (e.g., Malmodin et al. 2014), or representations of global network systems (e.g., Baliga et al. 2009). Estimates based on data for equipment specific to a certain service, as by Coroama and colleagues (2013), are limited and unlikely to give representative estimates for average transmission network electricity intensity.

Furthermore, studies should consider the full range of equipment in use within the network under study. This includes considering the legacy equipment within networks. Estimates based on specific or state-of-the-art equipment, such as Baliga and colleagues (2009), omit the less efficient legacy equipment (i.e., equipment with higher electricity use per GB of data transferred) in use within country-wide Internet networks, resulting in a substantial underestimate of electricity intensity at the lower end of the observed range (0.004 kWh/GB for 2008).

From this analysis of the methods used, the following criteria are identified:

1. The approach used should at least provide representative estimates of transmission networks at the national level.
2. Estimates should be based on data representative of the range of equipment deployed in national-level networks (i.e., including any legacy devices).

### Year to Which the Data Apply

Another important factor when considering existing estimates is the year to which the data apply. It is important that data underpinning an estimate are based on the same reference year; or, adjusted to represent the year under study, using reasonable and justified assumptions. Williams and Tang (2012) estimate the carbon intensity (from which we have calculated the electricity intensity) of data transmission for the year 2010, based on data for equipment from 2005. There appears to be no consideration for change in energy use of equipment from 2005 to 2010, which for multiple reasons presented below, could lead to inaccuracy in the final result.

As discussed previously, several estimates extrapolate older estimates and apply assumptions about the change in energy use, data traffic, or efficiency of the Internet over time. For example, an estimate for the year 2000 by Koomey and colleagues (2004) is based on data for AEC estimates of network equipment from Roth and colleagues (2002) (adjusted to account for cooling, ventilation, and auxiliary equipment). Taylor and Koomey (2008) subsequently corrected this estimate and derived estimates for 2006 by applying actual growth factors for equipment energy use from the U.S. Environmental Protection Agency (US EPA) (2007). Weber and colleagues (2010) later used the trend from 2000 to 2006 from Taylor and Koomey (2008), extrapolating to estimate the electricity intensity of data transmission for 2008.

Shehabi and colleagues (2014) also derive their estimate of electricity intensity for 2011 by applying energy efficiency improvement factors to the 2009 and 2010 estimates made by Coroama and colleagues (2013) and Malmodin and colleagues (2014), respectively. The problems with extrapolating results over time stem from the various contributions to variability: technology improvement, renewal of equipment, growth in usage, and major technological shifts.

#### Technology Improvement

It is difficult to measure the rate at which the power consumption of Internet technologies changes. Increased processing power of equipment has in the past followed Moore’s law, whereby every two years chip density doubles due to technological advances leading to increased number of transistors per unit area (Koomey et al. 2011). Increased processing power can lead to increased energy efficiency, as equipment is able to perform the same tasks with less energy expenditure (Koomey et al. 2011). Although Moore’s law has already slowed (Koomey and Naffziger 2015, 2016), the energy efficiency of technology is still expected to improve with gains expected from “improvements to circuit design, component integration, and software, as well as power-management schemes” (Koomey et al. 2014). While the constraints on networking equipment efficiency are somewhat different than those affecting general purpose computing devices, the broader trends identified by Koomey and colleagues (2011) and Koomey and Naffziger (2015, 2016) are suggestive of the rates of change we would expect to see in networking devices constructed from silicon microprocessors and related components.

#### Renewal of Equipment

The impact of new technology on the electricity efficiency of the network is dependent on the renewal rate, usually determined by the cost of amortization of capital equipment. Historically, the energy efficiency of computing equipment at peak output doubled every 1.6 years to the year 2000 (Koomey et al. 2011) and then doubled every 2.6 years after 2000 (Koomey and Naffziger 2015, 2016). Energy-use data for state-of-the-art equipment alone should generally not be used as a basis for calculations of electricity intensity of country-wide networks, because this will leave the energy cost of legacy equipment in the network (which is much less efficient than new equipment) uncounted, as is the case for the estimate of Baliga and colleagues (2009).

#### Growth in Data Flows

Data flows over Internet networks continue to grow rapidly as more people utilize the Internet and as population and data consumption per person increase. A white paper released by Cisco (2015) predicts Internet traffic growth of 42% per year to 2020. The increase in data use has also been coupled with increases in the number of connected devices, a trend that is likely to extend with the era of the “Internet of Things” (IEA 2014). This rapid growth requires ISPs to increase the capacity of networking infrastructure (Krug et al. 2014), which puts upward pressure on power consumption. As this growth is due to multiple factors, it is difficult to model and extrapolate, so such calculations should be closely tied to empirical evidence.

#### Major Technological Shifts

In addition, energy efficiency improvements can be hard to predict due to the potential for technology shifts that do not follow historical projections. Over long time periods, step changes in technology can be observed. For the Internet, this could be considered moving from technologies such as dial-up to ADSL broadband or more recently from ADSL broadband to fiber optic broadband, driven by demand for higher Internet speeds. Updating estimates by applying factors for changes in energy use, data traffic, or energy efficiency over time therefore should be done cautiously and with full knowledge of recent data on those trends.

The accuracy of any extrapolation will depend on the accuracy of predictions of trends in technology development, equipment deployed, usage, and technological shifts. Any extrapolation therefore must consider the potential of all these factors, making use of industry roadmaps, in addition to past trends. This leads to a third criterion:

3. If extrapolation is used, it should be based on analysis of planned future technological development and improvement over short periods (using industry roadmaps) rather than past trends alone.

### Access Networks

Access networks comprise many different types of equipment, highlighted in table 1. The bandwidth a customer receives depends largely on their access network, with Fiber to the node (FTTN) providing much higher average speeds than ADSL (Baliga et al. 2009), for example. In table 2, the access networks considered in each study range from specific, for example, based on FTTN only (Coroama et al. 2013), to inclusive of all access networks within national boundaries (Malmodin et al. 2014; Krug et al. 2014). Newer fiber optic access technologies, such as FTTN, can provide more efficient data transmission, with less electricity used per bit compared to older copper-based technologies (e.g., ADSL). An estimate for average electricity intensity should be inclusive of all access network types within the network under study. The fourth criterion is therefore:

4. Estimates must be based on data inclusive of all access network types within the network under study, based on data flows through each network in a given year.

### Technical Assumptions

Several technical assumptions are commonly used across the studies; these assumptions therefore are compared below in order to test their impact on the variability of estimates.

#### Utilization Factor

Utilization factor is the ratio of actual use to the total use capacity of a network. Values for utilization factor applied in the studies ranged from 15% (Schien et al. 2014) to 100% (Baliga et al. 2009). Choice of utilization factor is linked to the method used to derive the estimate. Comprehensive AEC studies and direct measurements based on organizational data do not require assumptions for utilization as the actual usage of networking equipment is implicit within the result.

**Figure 2:**
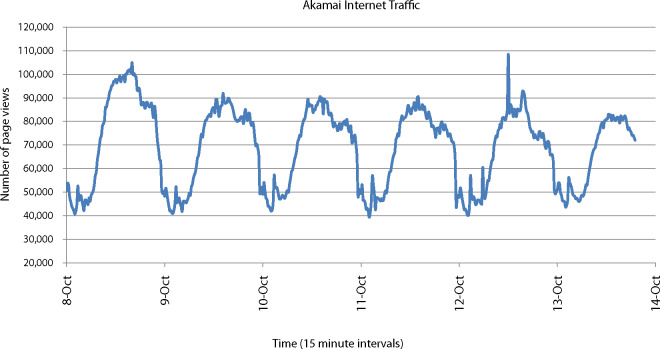
Example of daily variation of Internet traffic in 2012, based on number of page views per 15-minute interval for part of the Akamai network (Peill-Moelter 2012, reprinted with permission).

Internet networks at national scale exhibit diurnal usage patterns, with peak periods of activity occurring in the evening, as demonstrated in figure 2 (Peill-Moelter 2012).

ISPs provision networking infrastructure to provide bandwidth capacity for peak usage, so, for most of the day, networks are not utilized at maximum capacity. Some types of networking equipment, such as access network and home routers, do not typically scale energy use effectively with data traffic, consuming similar energy when in high and low use (Harrington and Nordman 2014). An assumption of 100% utilization is not representative of average transmission networks due to diurnal usage patterns and therefore can lead to underestimates of electricity intensity. Likewise, electricity consumption during underutilized times of day can be unaccounted for if estimates are based on transmission time alone. Williams and Tang (2012) follow this approach and their estimate is based on the product of equipment power consumption and transmission time. The electricity consumed to ensure the service can be provided at all times of the day, for example, is therefore not included. This could be a contributing factor to their estimate being an order of magnitude lower than Malmodin and colleagues’ (2014) estimate for the same year. In summary, lower values for utilization factor, such as used by Schien and colleagues (2014), are more likely to be representative of national-scale networks; this leads to the next criterion:

5. a) Estimates for utilization must reflect the average diurnal usage exhibited in networks, that is, not 100%.

#### Power-Use Effectiveness

Power-use effectiveness (PUE) is a measure of energy efficiency for network subsystem facilities, measured as the total energy used by the facility divided by the energy used by Information Techonology (IT) equipment (i.e., servers, routers, etc.). This factor provides a measure of energy efficiency of all equipment required in the system, including equipment not directly used to provide computation, such as power provision and cooling. Across 10 of the 14 studies, PUE ranges from 1.25 to 2.0. Shehabi and colleagues (2014) estimate PUE to be 1.3; this represents a specific example using an efficient equipment setup—the European Union (EU) code of conduct for data centers sets targets for best practice PUE of 1.2 or less (EC 2014). It is unlikely such low estimates of PUE represent the average for facilities within a national network.

Krug and colleagues (2014) and Malmodin and colleagues (2014) are able to verify estimates for average PUE by comparing modeling-based estimates, with empirical data for UK and Swedish networks, respectively. If PUE is a required assumption for estimates, we suggest a range for PUE of 1.8 to 2.0, as presented in these studies, appears representative for current typical Internet networks (although these values represent those typical of data centers and there is still uncertainty and further research required for estimating PUE of equipment in core/access networks). Lower values for PUE are possible for equipment used in specific services and average PUE of equipment in the Internet network may improve in the future.

- 5. b) Where PUE is a required assumption, average values should be between 1.8 and 2.0 in recent years (possibly higher for estimates for the early 2000s and lower for more advanced facilities).

#### Number of Hops

Number of hops is a measure of how many different nodes data pass through in the data transmission network. Values for number of hops ranged from 12 (Schien and Preist 2014) to 24 (Coroama et al. 2013) and is an assumption applied in 6 of the 14 studies. The relationship between the number of hops and the final intensity estimate is not as clear as that for utilization and PUE (which are multipliers) and varies between studies, depending on the specific model. Assumptions for number of hops could affect electricity intensity results; however, the magnitude of this effect is unclear.

It is difficult to measure the average number of hops for Internet use. Coroama and colleagues (2013) estimate hops for a specific service, while Krug and colleagues (2014) are able to corroborate their assumptions using BT organizational data for the entire UK network. If an assumption for number of hops is applied, estimates should be corroborated by empirical data representative of the whole system.

- 5. c) Estimates for number of hops should be corroborated by empirical data and be representative of data flows across the whole network.

Applying the criteria identified above to each study (table 3), the most representative estimates for the electricity intensity of transmission networks (i.e., excluding data centers and edge devices), shown in table 4, are: 6.5 to 7.1 kWh/GB for 2000 and 0.65 to 0.71 kWh/GB for 2006 (Taylor and Koomey 2008); 0.16 kWh/GB for 2010 (Malmodin et al. 2014), 0.14 kWh/GB for 2012 (Krug et al. 2014), and 0.023 kWh/GB for 2015 (Malmodin and Lundén 2016) .

**Table 4:** Final criteria and results from applying these criteria to each of the studies considered in this meta-analysis (highlighted columns denote those studies which satisfy all of the criteria)

Criterion		Koomey et al. (2004)	Taylor and Koomey (2008)	Baliga et al. (2009)	Weber et al. (2010)	Coroama et al. (2013)	Williams and Tang (2012)	Costenaro and Duer (2012)	Malmodin et al. (2012)	Malmodin et al. (2014)	Shehabi et al. (2014)	Schien and Preist (2014)	Krug et al. (2014)	Schien et al. (2014)	Malmodin and Lundén (2016)
1.	The approach used should at least provide representative estimates of transmission networks at national level.	✓	✓	✓	✓	✓	✓	X	X	✓	✓	✓	✓	✓	✓
2.	Estimates should be based on data representative of the range of equipment deployed in national-level networks (i.e., including any legacy devices).	✓	✓	X	✓	X	X	X	✓	✓	✓	X	✓	X	✓
3.	If extrapolation is used, it should be based on analysis of planned future technological development and improvement over short periods, (using industry roadmaps) rather than past trends alone.	n/a	n/a	X	X	n/a	X	n/a	n/a	✓	X	X	✓	n/a	n/a
4.	Estimates must be based on data inclusive of all access network types within the network under study, based on data flows through each network in a given year.	✓	✓	✓	✓	✓	✓	X	✓	✓	✓	✓	✓	X	✓
5a.	Estimates for utilization must reflect the average diurnal usage exhibited in networks, that is, not 100%.	n/a	n/a	X	n/a	X	X	✓	n/a	n/a	✓	n/a	n/a	✓	n/a
5b.	Estimates for PUE should be between 1.8 and 2.0 in recent years (possibly higher for specific estimates in the early 2000s).	✓	✓	✓	✓	X	✓	✓	✓	✓	X	✓	✓	✓	✓
5c.	Estimates for number of hops should be corroborated by empirical data and be representative of data flows across the whole network.	n/a	n/a	X	n/a	n/a	✓	X	n/a	n/a	X	X	✓	X	n/a

*Note*: n/a = not applicable.

Based on these results, trends in the electricity intensity of transmission networks and findings relating to methodology are discussed below.

## Discussion

**Figure 3:**
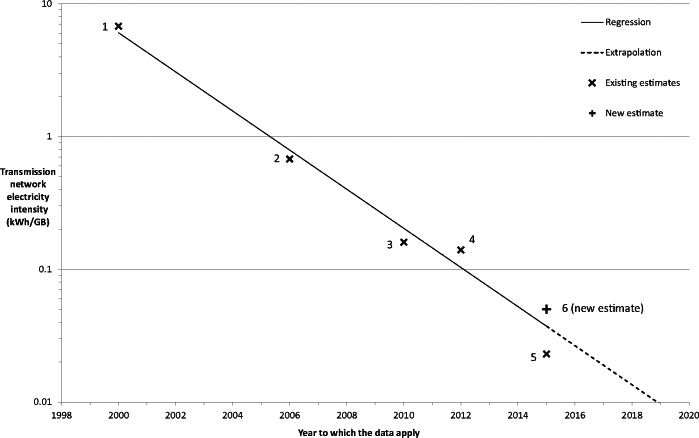
Graph to show estimates for electricity intensity for the transmission network system boundary only, identified from the criteria derived in this study. The y-axis shows the value of electricity intensity (kWh/GB) for each estimate; note the Log_10_ scale. The x-axis shows the year in which the data for each estimate is based. Regression uses average estimates for years in which a range is given and uses all data points on the graph from 2000 to 2015 (including our newly derived estimate for 2015). Data points: (1) median estimate of 6.5 to 7.1 kWh/GB derived from Taylor and Koomey (2008) estimates for the year 2000; (2) median estimate of 0.65 to 0.71 kWh/GB derived from Taylor and Koomey (2008) estimates for the year 2006; (3) estimate of 0.16 kWh/GB for 2010 derived from Malmodin and colleagues (2014); (4) estimate of 0.14 kWh/GB for 2012 derived from Krug and colleagues (2014); (5) Estimate of 0.023 kWh/GB from Malmodin and Lundén (2016); and (6) estimate of 0.06 kWh/GB for 2015 is a new estimate proposed in this study, based on Krug and colleagues (2014) with updated data for 2015 from Krug (2016). kWh/GB = kilowatt-hours per gigabyte.

For the five studies that satisfy our criteria, the electricity intensity of transmission networks has declined by factor of ∼170 between 2000 and 2015. Krug (2015) estimates that the electricity intensity of BT’s access networks has halved and core network intensity has declined by a factor of 10 from 2012 to 2015. Updating Krug and colleagues’ (2014) 2012 estimate using these assumptions gives a value for the electricity intensity of data transmission of 0.06 kWh/GB for 2015 (based on BT network in the UK). This estimate is similar to the updated estimate for 2015 from Malmodin and Lundén (2016). These results are displayed in figure 3, which shows the electricity intensity of data transmission over the period observed to halve approximately every 2 years (coefficient of determination, R^2^ = 0.98). Interestingly, this rate of improvement is somewhat faster than post-2000 historical trends in the electrical efficiency of computing at peak output observed by Koomey and Naffziger (2015, 2016).

Also shown is an extrapolation of the observed trend past 2015, demonstrating the potential for the reduction of transmission network electricity intensity if this trend continues with the same trajectory in the near future. Future research should continue to make original estimates that satisfy the criteria outlined in this study, as the extrapolated trend is based on limited data points and sensitive to the many variables discussed in previous sections. Nevertheless, this regression can be used to derive estimates of transmission network electricity intensity for all years between 2000 and 2015, where data may not be available from published studies.

Rather than using top-down or bottom-up methods, existing studies were found to use four distinct methods (or combinations of these) to estimate the electricity intensity of transmission networks; modeling, AEC, direct measurement, and extrapolation. The particular method used was not found to be a cause of much variability in estimates, as previously suggested. The variability observed in estimates can be attributed to differences in system boundary between studies and methodological errors including:

- Network studied not representative of entire Internet network in terms of scale or technical assumptions.
- Extrapolations based on past trends alone, rather than justified future predictions.
- Assuming 100% utilization is representative (in national-level networks utilization is <100%).
- Not including data for all types of fixed-line access networks.

For future research, in the case that the Internet network is considered an essential part of the system under study (the *foreground*), then more specific understanding may be required on drivers of increased electricity use and a *consequential* method of allocation (EC 2010) may be appropriate, for example, based on weighted averages or marginal changes in electricity use and data flow. Possible approaches to consequential allocation of electricity intensity are listed in table 5.

**Table 5:** Possible consequential allocation methods for Internet energy intensity

*Component*	*Possible allocation method*
Electricity used for Internet service provided	Time (h) × Power Consumption (W) × [Total Data Used (GB) / Total Capacity (GB)]
Electricity used to power unutilized data capacity equipment	Should be allocated in proportion to the share of peak data capacity a particular service uses at any point time

*Note*: W = watts; GB = gigabytes.

If networks were utilized at 100% capacity, allocation would be based on average electricity intensity for both consequential and attributional approaches. Electricity used directly to transmit data for a particular service over time therefore should be calculated as a function of time and data capacity used. Allocating electricity used to power the unutilized network capacity should then be distributed proportionally to those services requiring peak data capacity—since it is these services that drive ISPs to install additional capacity and bandwidth.

In future, networking equipment may scale its power consumption with different levels of utilization and also enter more power efficient idle modes when inactive (IEA 2014). Consequently, allocation methods must be continually updated to reflect changes in networking technology and energy performance. Future research could examine consequential versus attributional allocation for calculating electricity intensity of transmission networks in more detail.

## Conclusions

Existing estimates of Internet data transmission electricity intensity have varied greatly since 2000. Following Coroama and Hilty (2014), system boundary can be a significant cause of variation between estimates, together with the assumptions applied. Contrary to previous studies, our analysis did not find the methods used to be a substantial cause of variation between estimates; rather, the treatment of time, methodological errors, and boundary choices appear to be the major sources of uncertainty. To avoid common errors in the future, estimates of average transmission network electricity intensity should consider the criteria identified above.

Estimates for average transmission network electricity intensity that meet these criteria show a halving of intensity every 2 years. Our regression can be used to estimate Internet core and access network electricity use for each year between 2000 and 2015, helping to resolve previous uncertainty in this area. More research is required to update estimates for current and future years, and improve certainty of estimates and trends.

In addition, future work is needed to refine consequential methods of allocating the electricity intensity of transmission networks for use in special cases. Attributional allocation will likely remain the most pragmatic approach for use in LCA, so estimating average electricity intensity will remain a priority for research.

## Supplementary Information

**Supporting Information S1**: This supporting information provides additional information regarding the calculations made to convert existing estimates for Internet electricity intensity for the original studies system boundaries to a common system boundary of Internet core and access networks only (data transmission network).
